# Genome-wide association study for morphological, phenological, quality, and yield traits in einkorn (*Triticum monococcum* L. subsp. *monococcum*)

**DOI:** 10.1093/g3journal/jkab281

**Published:** 2021-08-23

**Authors:** Andrea Volante, Delfina Barabaschi, Rosanna Marino, Andrea Brandolini

**Affiliations:** 1 CREA—Research Centre for Cereal and Industrial Crops, 13100 Vercelli, Italy; 2 CREA—Research Centre for Genomics and Bioinformatics, 29017 Fiorenzuola d’Arda, Italy and; 3 CREA—Research Centre for Animal Production and Aquaculture, 26900 Lodi, Italy

**Keywords:** einkorn, wheat, genome-wide association study (GWAS), marker-trait associations (MTAs)

## Abstract

Einkorn (*Triticum monococcum* L. subsp. *monococcum*, 2*n* = 2× = 14, A^*m*^A^*m*^) is a diploid wheat whose cultivation was widespread in the Mediterranean and European area till the Bronze Age, before it was replaced by the more productive durum and bread wheats. Although scarcely cultivated nowadays, it has gained renewed interest due to its relevant nutritional properties and as source of genetic diversity for crop breeding. However, the molecular basis of many traits of interest in einkorn remain still unknown. A panel of 160 einkorn landraces, from different parts of the distribution area, was characterized for several phenotypic traits related to morphology, phenology, quality, and yield for 4 years in two locations. An approach based on co-linearity with the A genome of bread wheat, supported also by that with *Triticum urartu* genome, was exploited to perform association mapping, even without an einkorn anchored genome. The association mapping approach uncovered numerous marker-trait associations; for 37 of these, a physical position was inferred by homology with the bread wheat genome. Moreover, numerous associated regions were also assigned to the available *T. monococcum* contigs. Among the intervals detected in this work, three overlapped with regions previously described as involved in the same trait, while four other regions were localized in proximity of loci previously described and presumably refer to the same gene/QTL. The remaining associated regions identified in this work could represent a novel and useful starting point for breeding approaches to improve the investigated traits in this neglected species.

## Introduction

Plant breeding has significantly improved the production of many crops but has often exerted a bottleneck effect on the genetic diversity of several characters (Bedő and Láng 2015; Louwaars 2018). Modern germplasm, such as wheat, is highly unbalanced compared to the ancestral one found in landraces due to recent selection and spread. The characterization of these genetic resources is thus strategic to better exploit them efficiently into pre-breeding programs (Balfourier *et al.* 2019). The exploitation of ancient landraces or wild species requires in-depth phenotypic and genetic characterization to detect superior phenotypes and to identify their underlying genes and/or linked genetic markers (Liu *et al.* 2017; Zhou *et al.* 2017; Garcia *et al.* 2019; Rahimi *et al.* 2019; Kumar *et al.* 2020; Sansaloni *et al.* 2020). Germplasm banks currently host and preserve extensive collections of cultivars, landraces, and wild relatives of most crops, which are an excellent source of genetic variability for allele mining of important traits (Leung *et al.* 2015; Sehgal *et al* 2015; Brozynska *et al.* 2016; Zhang *et al.* 2016; Engels and Thormann 2020). The study of this largely untapped biodiversity would provide both the opportunity to recover new allelic variation, urgently required by breeding programs, and to work on the improvement of key traits directly in ancient materials.

Einkorn (*Triticum monococcum* L. ssp. *monococcum*) is a hulled diploid wheat (2*n* = 2× = 14) which was domesticated during the Neolithic Age in the Karacadağ mountain range (Turkey) from its wild ancestor *T.* *monococcum* L. ssp. *boeoticum* (Heun *et al.* 1997, 2008; Haldorsen *et al.* 2011). Einkorn quickly spread by different routes of migration throughout Europe (Brandolini *et al.* 2016), where it was widely used as human food for millennia before being gradually replaced, from the Bronze Age, by the polyploid wheats, characterized by higher production and free-threshing grains. Nowadays einkorn is cultivated in marginal regions and only recently, breeding efforts were started in several European countries (Hidalgo and Brandolini 2019).

Increased awareness on the nutritional properties of foods and recent trends toward low-impact and sustainable agriculture have led to a renewed interest for this ancient wheat. Einkorn composition and nutritive values are different, often better, from those of bread and durum wheat. For example, einkorn kernels are rich in proteins, lipids (mostly unsaturated fatty acids), fructan and trace elements, including Zn and Fe. Additionally, *T. monococcum* grains have good concentration of several antioxidant compounds (carotenoids, tocols, conjugated polyphenols, alkylresorcinols, and phytosterols) that, along with low beta-amylase and lipoxygenase activities (which limit antioxidants degradation during food processing), confer to the flour excellent nutritional benefits, superior to those of all the other wheats (Hidalgo and Brandolini 2019). Although not suitable for coeliacs (Zanini *et al.* 2015a,b), einkorn elicits weaker toxic reactions than other *Triticum* species (Mølberg *et al.* 2005; Vincentini *et al.* 2007; Di Stasio *et al.* 2020) and thus might be tolerated by patients with some types of food sensitivities (Lombardo *et al.* 2015).

The *T.* *monococcum* genome shows a high gene conservation and co-linearity with durum and bread wheat, even though the polyploid species inherited their A genome from *Triticum urartu* (Dubcovsky *et al.* 1996; Bai *et al.* 2004; Brandolini *et al.* 2006). Some einkorn genetic maps are available in literature (Dubcovsky *et al.* 1996; Kojima *et al.* 1998; Taenzler *et al.* 2002; Singh *et al.* 2007; Jing *et al.* 2009; Yu *et al.* 2017) and a high-resolution map based on Diversity Arrays Technology and sequencing (DArTseq) markers was recently developed (Marino *et al.* 2018). Nevertheless, only few data exist about the molecular basis of traits of interest in einkorn. The soft glumes (*Sog*, on 7L) trait, the loci coding for seed storage proteins, and Quantitative Trait Loci (QTL) for SDS sedimentation volume and protein content have been located by Taenzler *et al.* (2002). Alvarez *et al.* (2016) mapped the earliness-related locus *Eps-A*^m^ 1 in einkorn genome (on 1L) and identified EARLY FLOWERING 3 (*ELF3*) as the underlying candidate gene, while Chen *et al.* (2018) studied and mapped two stem rust resistance genes (*SrTm5* on 7L and *Sr60* on 5S). Very recently, Yu *et al.* (2019) analyzed grain size-related traits and identified the involved loci, by linkage and homology mapping and transcriptome profiling.

The new approach to genetic resources based on second generation sequencing techniques (genotype by sequencing, DArTseq) has paved the way to a sudden improvement of knowledge about the genomic regions involved in the control of traits of interest by genome-wide association studies (GWASs). However, the full exploitation of these genetic tools in einkorn has long been limited by the poor characterization of the available germplasm. Nonetheless, Jing *et al.* (2007) using association mapping characterized the phenotypic variation and identified several loci involved in the control of traits related to stress adaptation, phenology, and morphology of this species.

In this study, a panel of 160 *T.* *monococcum* ssp. *monococcum* landraces was subjected to association mapping analysis for complex agronomic traits related to phenology, morphology, yield, and quality. These accessions were genetically characterized with a DArTseq approach, which yielded two types of markers: (i) silicoDArT and (ii) SNP markers. Numerous marker-trait associations (MTAs), related to the traits analyzed, were identified; three of these overlapped with previously described loci, while four were located in proximity. The remaining associations represent mainly novel loci involved in these traits.

## Materials and methods

### Plant material, phenotyping, and statistical analysis

The panel used in this study consisted of 160 *T.* *monococcum* ssp. *monococcum* landraces, belonging to a core collection maintained at CREA in Sant’Angelo Lodigiano (Lodi, Italy). The set included material coming from different sites across Europe and neighboring areas (Morocco, Turkey, Near East and Caucasus). The accessions tested, selected mainly on passport data and previous research (Brandolini *et al.* 2016; Volante et al. 2020), represent a well-balanced sampling of current *T. monococcum* distribution and diversity (Heun *et al.* 1997; Empilli *et al.*, 2000). Several accessions for each geographic location were included, depending on availability, as well as selections with good bread making quality. This latter was assessed by micro tests (SDS sedimentation test; Preston *et al.*, 1982), Brabender farinograph and bread baking according to method 10–10.3 (AACC International 2012a). For further description and details, see Supplementary Table S1, Brandolini *et al.* (2008) and Volante et al. (2020). The field trials were performed in two locations (Sant’Angelo Lodigiano: 45°14′21″N 9°24′22″E, and Lodi: 45°18′12″N 9°30′45″E) for 4 years (from 2010–2011 to 2013–2014). About 50 plants per accession were planted in one single 2 m row for each environment. Standard cultural practices were applied, including nitrogen fertilization (40 kg/ha) at tillering and chemical weed control (Ariane II: Fluroxipir + Clopiralid + MCPA). The spikes were manually harvested at full maturity and seeds stored at 5°C. Whole meal flour was prepared from 10 g de-hulled kernels using a Cyclotec 1093 laboratory mill (FOSS Tecator, Denmark) and stored at −20°C until analysis.

The landraces were characterized for thirteen morpho-agronomic descriptors: heading date (HDA, number of days, starting from May 1st, when about 50% of the plants shows emerging spikes), plant height (PLH, excluding awns, in cm), awn length (AWN, from 1 = no awns to 5 = very long awns, *i.e.*, longer than spikes), glume color (GLC, 1 = white-cream, 2 = brown, 3 = black, 4 = two colors), and glume hairiness (GLH, 1 = no hair, 2 = short, sparse, 3 = short, thick, 4 = long, thick), rachis brittleness (RAB, 1= very brittle to 5 = non-brittle), spike length excluding awns (SPL, in mm), n° spikelets/spike (SPS), n° kernels/spikelet (GPS), grain length (GRL, in mm), grain width (GRW, in mm), grain thickness (GRT, in mm), and thousand grains weight (TGW, computed from two 100-kernel samples). The data were collected from 10 plants, 10 spikes, or 20 kernels for each landrace. Additionally, three quality traits were assessed: protein content (PRO, N × 5.7, dry matter basis) determined on whole meal according to method 46-10.01 (AACC International 2012b), using a NIR System Model 6500 (FOSS NIRSystems, Laurel, MD, USA), sodium dodecyl sulphate sedimentation volume (SDS) recorded according to Preston *et al.* (1982), and carotenoid content (CAR) assessed following method 14-60.01 (AACC International 2012c) using a DU-62 spectrophotometer (Beckman Coulter Inc., Brea, CA, USA). The qualitative descriptors were measured from at least two technical replicates.

An analysis of variance (ANOVA) was conducted throughout the set of phenotypic data, using genotypes and years as factors; the locations were used as repetitions. Broad sense heritability (H^2^) was calculated according to Nyquist (1991):

$$H^{2}=\sigma^{2}{}_{G}/\left[\sigma^{2}{}_{G}+\left(\sigma^{2}{}_{\text{GE}}/E\right)+\left(\sigma^{2}{}_{e}/\text{rE}\right)\right]$$

where σ^2^_G_ is the genetic variance, σ^2^_GE_ is the genotype × environment interaction variance, σ^2^_e_ is the residual variance, E is the number of environments, and r the number of replicates. All analyses were performed using the STATGRAPHICS^®^ Centurion XV (Statgraphics Technologies, Inc., The Plains, VA, USA) statistical program.

### Genotypic data and *in silico* mapping

The genotypic data for the 160 accessions consisted of a set of 33,260 DArTseq markers, described in Brandolini *et al.* (2016), including both SNP and SilicoDArTs. DNA extraction from 7-day-old seedlings (one plant per accession) was performed with a standard CTAB protocol as described by Stein *et al.* (2001). DNA concentration was quantified with a NanoDrop ND-1000 spectrophotometer (Thermo Fisher Scientific Inc., Waltham, MA, USA). The DNA was analyzed with the DArT-seq technology (Kilian *et al.*, 2012) by the company Diversity Arrays Technology (Canberra, Australia). Twelve genotypes (*i.e.*, the very same DNA) were fingerprinted twice (unknown to the lab in Australia). The original panel was filtered for callrate and minor allele frequency (MAF) using the PLINK software (Purcell *et al.* 2007). Markers with a callrate lower than 90% and with MAF lower than 5% were discarded.

An einkorn physical map was produced *in silico* using the most exhaustive and comprehensive genome available for wheats so far, that of *Triticum aestivum*. A physical position of DArTseq markers on bread wheat genome was determined by aligning the corresponding nucleotide sequences (https://www.diversityarrays.com/technology-and-resources/sequences/), supplied by Diversity Array Technologies, to the A genome of the RefSeq v1.0 reference genome (IWGSC 2018). The einkorn genetic map of Marino *et al.* (2018) was used to orient the physical map developed in this work. The physical positions of 184 markers in common between these two maps were exploited as a scaffold (Supplementary Table S2).

A link to the *T. monococcum* contigs and to *T. urartu* pseudomolecules was also acquired blasting the same marker reads against TGAC WGS monococcum v1 (available at https://wheat-urgi.versailles.inra.fr) and against GCA_003073215.1 assembly (Ling *et al.*, 2018), respectively. BLASTN searches were performed using the tool available at the URGI (https://wheat-urgi.versailles.inra.fr/Seq-Repository/BLAST) for *T. aestivum and monococcum* genome; while for *T. urartu* BLAST was conducted locally on downloaded genome. All the analyses were done using BLASTN with these cutoffs: a minimum coverage of 99% (only one gap is admitted) and an identity over or equal to 97% (no more than two mismatches).

### Analysis of population structure

The analysis of genetic stratification in the einkorn panel was performed with two different methods: a principal component analysis (PCA) by the Tassel v5.2.0 software (Bradbury *et al.* 2007) and a phylogenetic clustering obtained utilizing the MEGA7 software (Kumar *et al.* 2016) with the neighbor-joining method and default parameters.

A Bayesian model-based analysis was achieved using the program Structure v2.3.4 (Pritchard *et al.* 2000). The parameters used were the following: presence of admixture, allele frequencies correlated, a burn-in period of 10,000 iterations, followed by 20,000 Monte Carlo Markov Chain (MCMC) replications, K levels from 1 to 10 and 5 runs per K-value. The free software Structure Harvester (Earl and von Holdt 2012) was used to determine the best number of clusters (K) according to the Evanno method. Once defined the most probable K-value, a final single run was performed using the same parameters listed above, but with a higher number of burn-in and MCMC iterations (100,000 and 200,000, respectively). Accessions with a 0.7 minimum membership (*i.e.*, the probability of one individual to belong to a subgroup identified by Structure) were assigned to a subpopulation, while the remaining were considered as admixed. The number and composition of the clusters identified were integrated into the results of the neighbor-joining analysis and the PCA for cross-validation.

### Analysis of linkage disequilibrium decay

The computation of pairwise linkage disequilibrium (LD; *r*^2^) was conducted as in Volante *et al.* (2017). Briefly, the whole set of markers was analyzed with the R package “LDcorSV v1.3.1” (Mangin *et al.* 2012), using as covariates the Structure membership matrix and the kinship matrix calculated by Tassel. The values were binned in 10 kb windows as in Biscarini *et al.* (2016).

For each of the nine linkage groups (*i.e.*, all the chromosomes including the split 2 and 4), the median value of each 10-Kb interval was calculated (this was used instead of the mean to account for a non-normal distribution of LD values at short and long-distance classes). The resulting values were plotted against physical distance and fitted to a second-degree LOESS curve using an R script (Cleveland 1979; Marroni *et al.* 2011). A critical value of 0.2 was set as *r*^2^ between unlinked loci. The physical distance corresponding to a LOESS curve value of 0.2 was assumed as LD decay in the einkorn panel.

### Association analysis

For the genome-wide association analysis, to combine data from multiple environments, the Best Linear Unbiased Predictors (BLUPs) of each trait for each accession were calculated using the lmer function of the lme4 R package (Bates *et al.*, 2015). The model fitted to the data considered genotype, year, and genotype: year interaction as random factors. The ranef function was used to calculated BLUPs, which were then used for the association analysis. To identify the best covariates for each trait, we used the method described by Courtois *et al.* (2013), *i.e.*, a preliminary GWAS was performed with Tassel, testing a Mixed Linear Model (MLM) corrected with (1) the kinship matrix and (2) the kinship matrix and 5 components of the PCA analysis. In all cases, the Tassel default options (“optimal compression” and P3D mode) were used. The best model was identified for each trait by calculating the Bayesian Information Criterion (BIC) as in Schwarz (1978); the BIC was computed as -2 ln(L) + kln(n) where L is the maximized value of the likelihood function for the estimated model (provided by the GWAS analysis), k is the number of estimated parameters and n is the size of the sample. The model with the lowest BIC value was selected. The final GWAS was then performed with the most appropriate model for each trait, with Tassel using the “no compression” and “no P3D” mode to increase the power of detection (Courtois *et al.* 2013). A *P*-value of the association to the phenotypic traits was computed for each marker; the significance threshold to declare a marker as associated was set to 0.05 after correction for multiple testing, using the false discovery rate method (Benjamini and Hochberg 1995). Manhattan plots and Q–Q plots of each trait were drawn using the R package “qqman” (Turner 2014).

## Results

### Statistical analysis of phenotypic results

The ANOVA conducted using year and genotype as factors indicated that both had significant effects on the phenotypic variation (Supplementary File S1), highlighting the strong effect of changing yearly climatic conditions and the broad variation available in the einkorn pool tested. This last aspect is evident in the data from Table 1, reporting the average, minimum and maximum values of the traits analyzed, expressed as average of four growing seasons (2011–2012, 2012–2013, 2013–2014, and 2014–2015) and two locations (SAL and LO), as well as their broad-sense heritabilities. These latter had remarkably high values, ranging between 0.896 (GLC) and 0.981 (HDA).

**Table 1:** Mean, minimum, maximum values, and broad-sense heritabilities (H^2^) for 16 variables measured on the 160 einkorn accessions

Trait	Mean	Minimum	Maximum	H^2^
HDA	28.17	10.25	38.50	0.981
PLH	108.89	47.66	126.88	0.931
AWN	3.90	1.88	4.50	0.947
GLC	1.35	1.00	3.00	0.896
GLH	1.11	1.00	2.50	0.948
GPS	1.08	0.85	1.44	0.902
GRL	7.39	6.61	8.24	0.953
GRT	1.93	1.37	2.22	0.915
GRW	2.88	2.41	3.31	0.922
RAB	3.71	3.13	4.38	0.923
SPL	7.20	3.70	9.70	0.933
SPS	28.43	23.24	34.84	0.943
TGW	27.20	16.05	35.66	0.952
CAR	8.18	4.92	11.46	0.967
PRO	17.11	14.36	20.70	0.924
SDS	19.19	11.63	56.61	0.959

AWN, awns length (from 1 = no awns to 5 = very long awns, *i.e.*, longer than spikes); CAR, carotenoids content (mg/kg dry weight); GLC, glume color (1 = white-cream, 2 = brown, 3 = black, 4 = two colors); GLH, glume hairiness (1 = no hair, 2 = short, sparse, 3 = short, thick, 4 = long, thick); GPS, N° kernels/spikelet; GRL, Kernel length (mm); GRT, Kernel thickness (mm); GRW, Kernel width (mm); HDA, heading (number of days); PLH, plant height (cm); PRO, proteins content (% dry weight); RAB, rachis brittleness (from 1 = very brittle to 5 = non-brittle); SDS, SDS sedimentation (mL); SPL, spike length (mm); SPS, N° spikelets/spike; TGW, thousand kernel weight (g).

### Using wheat genomes to define marker location

After filtering for missing data and MAF that eliminated 61.7% of the data, 12,734 out of 33,260 einkorn DArTs were retained for subsequent analyses (Supplementary File S2).

An einkorn physical map was developed *in silico* assigning to 2037 markers a physical position on the bread wheat reference genome (IWGSC RefSeq v1.0). The genetic map recently published (Marino *et al.* 2018) was employed as a scaffold to correctly build and orient the einkorn physical map. Two portions, one for two and one for four chromosomes, showed a marker order conserved but inverted respect to bread wheat genome. Therefore, these chromosomes were split in two portions each (“up” and “down”): the distal portion of chromosome 2 was inverted respect to the physical map of *T. aestivum* (chr 2_up: from 500,610 to 756,929,079 bp and chr 2_down from 780,777,947 to 756,949,663 bp), while in the case of chromosome 4, the two portions were swapped (chr 4_up from 610,263,195 to 738,781,733 bp and chr 4_down from 2,411,431 to 608,561,225 bp of bread wheat respectively; Supplementary Table S2). The number of DArT markers was highest in chromosome 2 (2_up plus 2_down) (386), while chromosome 4 (4_up plus 4_down) had the lowest number (133) (Supplementary Table S2). Mapped DArTs included 16% of the dataset.

Sequences of DArT markers were also aligned against the recently released version of *T. urartu* genome (Ling *et al.*, 2018): 1673 markers, corresponding to 13% of the whole dataset, were linked to a physical position using the same BLAST criteria adopted with bread wheat genome, with a number of markers/chromosome ranging between 107 (Chromosome 4) and 342 (Chromosome 2; Supplementary Table S4_a).

Moreover, following the same pipeline, a link to 3290 einkorn contigs (TGAC WGS monococcum v1) was acquired for 3655 markers (Supplementary Table S4_b).

### Population structure

**Figure 1:**
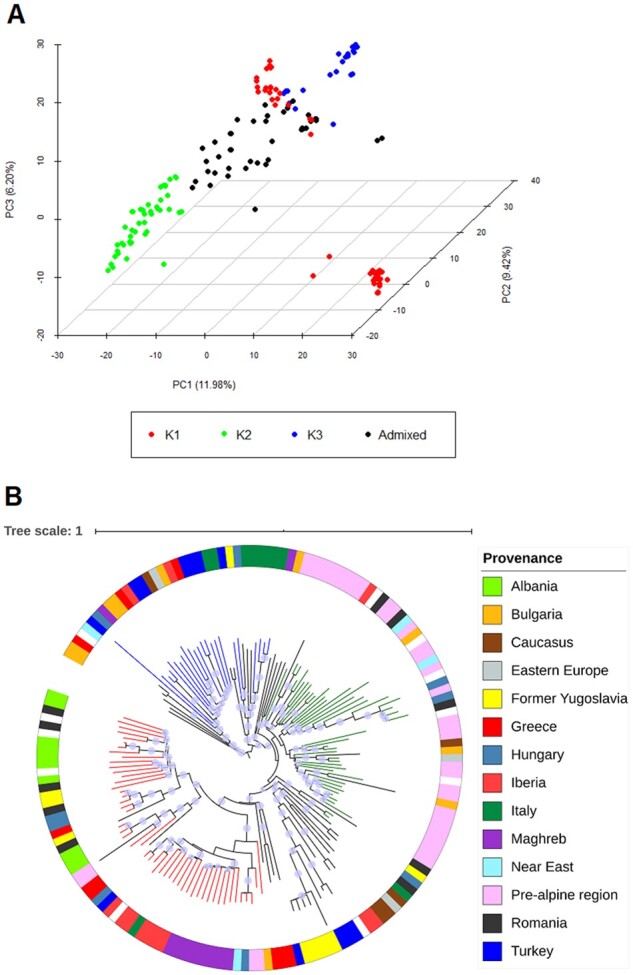
Analysis of population structure. (A) Principal component analysis (PCA) (the clusters in the legend are those defined by the STRUCTURE analysis); (B) neighbor joining tree. Red = mixed accessions originated from the Maghreb to the Balkans; green = mainly prealpine accessions; blue = mainly landraces from Turkey, Greece, and Italy. The blue-shadowed circles on each branch of the neighbor joining tree show the results of the bootstrap analysis with 1000 iterations, when higher than 0.7. The information about geographic provenance are also reported and color-coded (outer circle).

The results of the Principal Component, Structure and Neighbor Joining analyses are shown in Figure 1 and Supplementary Table S1. The most probable Structure model assigned 118 accessions (corresponding to 73.3% of the panel) to 3 clusters (Supplementary Table S1): K1, containing accessions of mixed origin from the Maghreb to the Balcans (red cluster), K2 which mainly included continental accessions (green cluster), and K3 composed mainly of landraces from Turkey, Greece, and Italy (blue cluster). The first three PCs accounted for 27.6% of the variability in the panel (Figure 1A). The clustering coming from PCA was also consistent with that resulted from the neighbor joining tree (Figure 1B); by crossing the results from the three analyses it was evident that the two groups separated by the PC3 in the K1 cluster (red cluster, Figure 1A), corresponded to the accessions from Maghreb and Spain, and those from East-European respectively (Figure 1A). This clustering was consistent with that previously proposed by Brandolini *et al.* (2016), which in this panel of landraces observed two groups splitting from the main cluster, one of Iberia-Maghreb and one of “prealpine” accessions (mainly from Austria, Germany, and Switzerland).

### Analysis of genome-wide linkage disequilibrium decay

**Figure 2:**
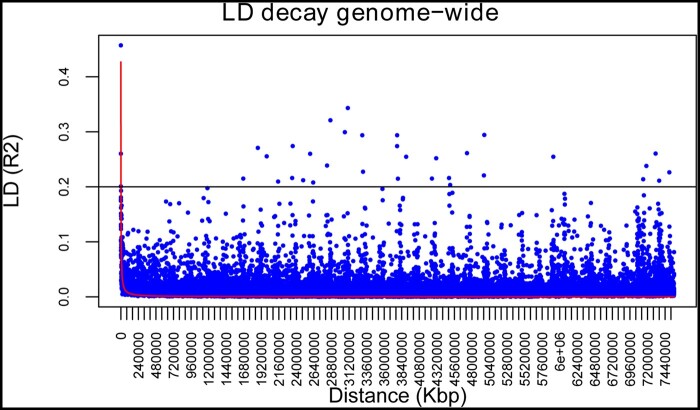
Genome-wide LD decay in einkorn accessions. The red curve represents the second grade LOESS that approximates the *r*^2^ values; the horizontal black line shows the threshold *r*^2^ value for unlinked markers.

The mean chromosome-wise decay of linkage disequilibrium, calculated as *r*^2^, was approximately 176 Kbp (Table 2). A heterogeneous distribution was observed over the different chromosomes, ranging from 25 kbp (chr. 6) to 395 Kbp (chr. 5). The average decay plot across all the chromosomes is shown in Figure 2.

**Table 2:** Analysis of chromosome-wise LD decay

Chromosome-wise LD decay	
Chr.	Critical decay distance^*a*^ (bp)
1	245,000
2_up	55,000
2_down	195,000
3	125,000
4_up	325,000
4_down	135,000
5	395,000
6	25,000
7	85,000
mean	176,111

a Corresponding to *r*^2^ values of 0.2.

### Association analyses

**Figure 3:**
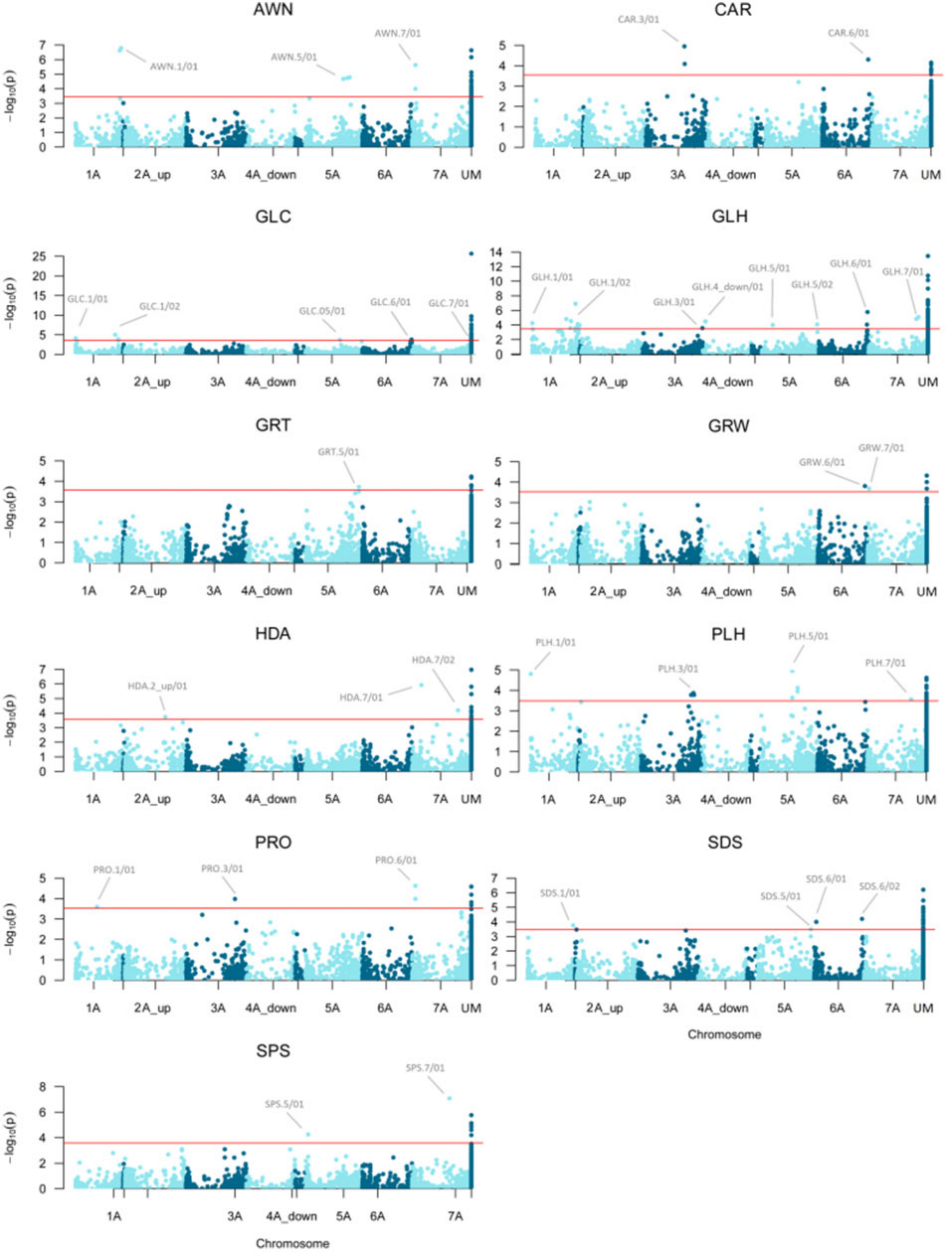
Manhattan plots showing the mapped marker-trait associations for the considered traits. UM = unmapped. The red line represents the significance threshold of 0.05 adjusted for the False Discovery Rate with the Benjamini–Hochberg test (see Materials and Methods for details).

In order to account for the interaction genotype × environment on the variability of the traits, association analyses were conducted on BLUP predictors calculated for each accession. The Manhattan and QQplot for each analysis are shown in Figure 3 and Supplementary File S3.

A total number of 64 markers with a reliable position on the *T. aestivum* genome (“mapped”) were associated with the analyzed traits, and defined 37 regions (Figure 3, Table 3), which were identified as trait acronym.chr number/region number. Furthermore, 274 associated but “unmapped” markers were identified (Supplementary Table S3).

**Table 3:** Results of the association analysis: mapped marker-trait associations (MTAs)

Results of the genome-wide association mapping analysis												
Trait category	Trait	MTA ID	Physical position (bp)^*a*^							Peak marker		
			Chr.	Start	End	Size		ID	Position	Alleles	MAF	*R* ^2^ ^*b*^
Morphology	AWN	AWN.1/01	1	549,283,004	566,163,941	16,880,937	SIL_1218444	566,163,941	pres/abs	6.00%	0.17282
Morphology	AWN	AWN.5/01	5	473,321,250	551,446,712	78,125,462	SIL_1018217	551,446,712	pres/abs	10.26%	0.11534
Morphology	AWN	AWN.7/01	7	41,818,199	41,877,671	59,472	SNP_3574217	41,877,671	66:A>G	17.95%	0.13713
Morphology	GLC	GLC.1/01	1	—	—	—	SIL_4409943	1,846,753	pres/abs	28.66%	0.09793
Morphology	GLC	GLC.1/02	1	492,800,956	530,809,593	38,008,637	SNP_2243185	492,800,956	58:T>C	5.16%	0.12221
Morphology	GLC	GLC.5/01	5	—	—	—	SIL_1281357	430,959,218	pres/abs	8.90%	0.09406
Morphology	GLC	GLC.6/01	6	—	—	—	SIL_1202445	611,334,867	pres/abs	10.19%	0.08933
Morphology	GLC	GLC.7/01	7	—	—	—	SIL_1067890	724,946,619	pres/abs	8.86%	0.08346
Morphology	GLH	GLH.1/01	1	—	—	—	SIL_1723678	1,674,029	pres/abs	25.32%	0.10053
Morphology	GLH	GLH.1/02	1	423,932,005	584,961,270	161,029,265	SNP_100057721	537,093,899	25:G>C	5.03%	0.16706
Morphology	GLH	GLH.3/01	3	—	—	—	SIL_1267588	743,193,869	pres/abs	8.13%	0.08229
Morphology	GLH	GLH.4_down/01	4_down	33,622,131	34,765,949	1,143,818	SIL_1202938	33,646,546	pres/abs	5.00%	0.106
Morphology	GLH	GLH.5/01	5	—	—	—	SIL_979056	135,162,954	pres/abs	5.77%	0.0956
Morphology	GLH	GLH.5/02	5	—	—	—	SIL_1122236	686,095,861	pres/abs	6.25%	0.09506
Morphology	GLH	GLH.6/01	6	596,658,016	603,182,559	6,524,543	SNP_1190777	603,182,559	22:A>G	5.73%	0.1403
Morphology	GLH	GLH.7/01	7	592,905,438	619,036,444	26,131,006	SNP_3572864	619,036,444	15:T>C	5.77%	0.12362
Morphology	GRT	GRT.5/01	5	—	—	—	SIL_2247447	665,892,637	pres/abs	38.82%	0.09051
Morphology	GRW	GRW.6/01	6	—	—	—	SIL_3572989	585,787,274	pres/abs	38.16%	0.09269
Morphology	GRW	GRW.7/01	7	—	—	—	SIL_1081679	20,582,384	pres/abs	13.92%	0.08641
Morphology	PLH	PLH.1/01	1	—	—	—	SIL_1092630	1,673,894	pres/abs	6.96%	0.11749
Morphology	PLH	PLH.3/01	3	621,592,828	658,048,755	36,455,927	SNP_2293071	649,815,961	68:G>A	6.29%	0.09278
Morphology	PLH	PLH.5/01	5	395,852,119	461,901,240	66,049,121	SIL_1204105	395,852,119	pres/abs	23.42%	0.12161
Morphology	PLH	PLH.7/01	7	—	—	—	SNP_994119	545,935,056	20:T>C	5.66%	0.08454
Morphology	SPS	SPS.5/01	5	—	—	—	SIL_1063095	36,199,250	pres/abs	16.35%	0.09316
Morphology	SPS	SPS.7/01	7	—	—	—	SNP_996521	464,570,003	16:A>G	5.03%	0.15418
Phenology	HDA	HDA.2_up/01	2_up	—	—	—	SNP_3949514	501,317,542	59:C>G	35.44%	0.08451
Phenology	HDA	HDA.7/01	7	—	—	—	SIL_5003498	115,960,770	pres/abs	15.65%	0.14264
Phenology	HDA	HDA.7/02	7	—	—	—	SIL_5006335	571,054,862	pres/abs	38.71%	0.09722
Quality	CAR	CAR.3/01	3	483,483,183	483,483,953	770	SNP_981918	483,483,183	19:G>A	9.32%	0.11867
Quality	CAR	CAR.6/01	6	—	—	—	SIL_4994928	572,312,243	pres/abs	23.33%	0.10336
Quality	PRO	PRO.1/01	1	—	—	—	SIL_3575496	266,740,723	pres/abs	6.29%	0.08065
Quality	PRO	PRO.3/01	3	—	—	—	SIL_1125220	608,744,656	pres/abs	37.34%	0.09107
Quality	PRO	PRO.7/01	7	41,818,199	41,877,671	59,472	SNP_3574217	41,877,671	66:A>G	17.95%	0.1077
Quality	SDS	SDS.1/01	1	—	—	—	SNP_100018797	568,008,907	5:A>T	6.21%	0.08513
Quality	SDS	SDS.5/01	5	—	—	—	SNP_1025773	665,609,700	27:A>G	5.19%	0.08069
Quality	SDS	SDS.6/01	6	—	—	—	SIL_3575693	25,706,107	pres/abs	10.76%	0.09259
Quality	SDS	SDS.6/02	6	—	—	—	SNP_1088712	593,787,612	44:A>G	5.66%	0.09513

a On IWGSC RefSeq v1.0.

b Phenotypic variance explained by the peak marker.

For morphology-related traits (AWN, GLC, GLH, GRL, GRW, PLH, and SPS), the analysis detected 25 associated regions with a defined position on the bread wheat genome. The percentage of variance explained by the peak markers (*r*^2^) ranged between 8.23% and 17.28%. GLH was the trait with the highest number of MTAs (eight mapped regions).

The GWAS on the phenology-related HDA resulted in three associated mapped regions (variance explained ranging between 8.45% and 14.26%), while for quality traits (CAR, SDS, and PRO) the analysis yielded in total nine associated regions (two for CAR, four for SDS, and three for PRO). The variance explained ranged between 7.8% and 15.7%.

For yield-related traits, one unmapped marker (SIL_3028492) resulted associated to TGW, with an explained variance of 9.0% (Supplementary Table S4_c). Physically overlapping regions associated to different traits were also detected, e. g. AWN.7/01 and PRO.7/01 completely overlapped, also sharing the same peak marker (SNP_3574217), while GLC.5/01 partially overlapped with PLH.5/01.

## Discussion

The renewed interest for cultivated einkorn, for millennia cropped in isolated areas of Middle East, Caucasia, and Europe, is centered on the nutritional qualities of its grain (Hidalgo and Brandolini 2019), the high resistance to pests and diseases (Zaharieva and Monneveux 2014; Serfling *et al.* 2016; Chen *et al.* 2018), and its adaptation to harsh, low-input environments. Low yields, glumes persistence after harvest (hulledness) and a limited knowledge of its diversity explain why this crop is not properly exploited (Zaharieva and Monneveux 2014).

Nevertheless, einkorn landraces were selected and grown in isolated environments and were not subjected to the “bottleneck” effects of intensive selection, thus even today display a broad range of variation for many traits. The panel of landraces used in this work was recently characterized from the phenotypic point of view (Volante et al. 2020); a deeper investigation of the genetic bases underlying such diversity may unravel valuable genes, potentially useful for wheat improvement. Linkage and association mapping rely necessarily on the availability of a great number of molecular markers with known genetic or physical position; a high-resolution einkorn genetic map was recently published (Marino *et al.* 2018), but only few markers (849) are in common with those included in the panel used in this work. By exploiting the high co-linearity of the genomes of *T. monococcum* and *T.* *urartu* (Marino *et al.* 2018), the latter being the donor of the A genome to bread wheat, we aligned the sequences of the DArT markers from a panel previously developed and described (Brandolini *et al.* 2016) on the annotated genome of bread wheat (IWGSC, 2018), being this latter the most exhaustive and comprehensive genome available for wheats so far. In such a way, a physical position could be approximated for 2037 out of the 12,734 markers used for the GWAS analysis (corresponding to 16% of the panel). We decided to use very stringent parameters for the BLAST analysis, despite knowing that we would lose most of the alignments, but due to the genetic distance between the two species (Hyun *et al.* 2020), we wanted to be as rigorous as possible and to give very robust physical localizations. In the exploitation of *T. urartu* genome the percentage of physically mapped markers was lower (13%: 1673) but the marker order along chromosomes of einkorn physical map was conserved with that of *T. aestivum* genome (Supplementary Table S4_a). These observations support our choice to use the bread wheat genome as the most appropriate tool to give a physical order to einkorn markers.

The association analysis, corrected for the population structure, was therefore undertaken using the markers thus physically mapped, together with those with unknown position.

The pairwise LD (*r*^2^) observed in the panel decayed to values lower than 0.2 (generally assumed as a *r*^2^ value between unlinked loci) at 176.11 Kbp (as an average value across all the chromosomes). Such an *r*^2^ is notably small, compared to what is generally observed in wheat species such as bread wheat (8 Mbp as an average among A, B, and D genome; Liu *et al.* 2017), and durum wheat (from 1.6 Mb to 4.5 Mb; Maccaferri *et al.* 2019), while for rice similar values have been observed (150 Kb to 2 Mb for the *japonica* group; Courtois *et al.* 2013; Kumar *et al.* 2015). Strongly selected crops typically show high levels of LD, due to increased correlation among alleles at specific loci (Schlötterer 2003); conversely, a short decay can be expected in a panel including landraces, which usually have a long history of isolated cultivation and were not or were poorly subjected to breeding. Accordingly, Hao *et al.* (2011) detected a lower LD in bread wheat landraces compared to modern cultivars; therefore, the results observed are not surprising because einkorn has a completely different history of domestication from “more successful” crops. However, it is remarkable that a significant percentage of the accessions used in this work (26.7%) were not assigned to a specific cluster by the STRUCTURE analysis, suggesting that a certain percentage of admixture was present.

The marker set used in this work (composed of 12,734 DArTseq) allowed to identify a high number of loci associated to the traits analyzed. We investigated whether the significantly associated regions (the interval defined by the peak marker ± 176 kbp, according to the extent of the LD decay) found in this study were located at the same or close physical position of previously identified genes or QTL with a known function.

The regions HDA.07/01 identified by marker SIL_5003498 (115.9 Mb) and HDA.07/02 identified by SIL_5006335 (571.1 Mb) were consistent respectively with two QTL located on Chromosome 7 at 119.5 Mb (AX-94419768) and at 576.1–581.2 Mb (qHD7A.4) reported previously in bread wheat (Turuspekov *et al.*, 2017; Pang *et al.*, 2020) (Table 4). For plant height, the region PLH.07/01 detected by SNP_994119 on Chromosome 7 at 545.9 Mb was previously identified by Pang *et al.* (2020) as qPH7A.5 (3.6-3.7 Mb) (Table 4).

**Table 4:** Comparison between the marker-trait associations (MTAs) detected in this work and QTL from literature

Correspondence of MTAs detected in this work with QTLs from literature								
Trait	MTA ID	Peak marker	Chromosome	Position (bp)^*a*^	QTL	Position	Distance (Mb)	Reference
**HDA**	**HDA.7/01**	**SIL_5003498**	**7**	**115,960,770**	**AX-94419768**	**119.5 Mb**	**3.6**	**Turuspekov et al. (** 2017)
**HDA**	**HDA.7/02**	**SIL_5006335**	**7**	**571,054,862**	**qHD7A.4**	**576.1–581.2 Mb**	**6**	**Pang et al. (** 2020)
**PLH**	**PLH.7/01**	**SNP_994119**	**7**	**545,935,056**	**qPH7A.5**	**539.9–540 Mb**	**5**	**Pang et al. (** 2020)
GLH	GLH.1/02	SNP_100057721	1	537,093,899	AX-94618537	511.1 Mb	26	Sheoran et al. (2019)
SPS	SPS.7/01	SNP_996521	7	464,570,003	qSNS7A.2	433.3–433.6 Mb	31	Pang et al. (2020)
SDS	SDS.5/01	SNP_1025773	5	665,609,700	AX-94927055	697.7 Mb	32.1	Yang et al. (2020)
PRO	PRO.3/01	SIL_1125220	3	608,744,656	AX-95629522	649.7 Mb	41	Yang et al. (2020)

For each known QTL considered, the position and distance from the corresponding MTA are reported. Boldface MTAs are located at <10Mb from the cited QTL reported in literature.

a On IWGSC RefSeq v1.0.

A survey of the literature suggests that the regions identified on chromosomes 3 (608.8 Mb) PRO.03/01 and 5 (665.6 Mb) SDS.05/01 are in proximity of (although not overlapping) those identified by Yang *et al.* (2020) in very recent multi-locus GWAS (at 649.7 Mb for grain protein content—AX-95629522; and at 697.7 Mb for SDS-sedimentation volume—AX-94927055) (Table 4).

The same considerations can be done for GLH on chromosome 1 (537.1 Mb; GLH.01/02) and for SPS on chromosome 7 (464.6 Mb; SPS.07/01) that appear to be in the proximity to regions associated to, respectively, glume pubescence at 511.1 Mb—AX-94618537 (Sheoran *et al.*, 2019) and spike/spikelet number at 433.3 Mb (Pang *et al.*, 2020) (Table 4).

The two regions identified for AWN on chromosome 5 (522.1 and 551.4 Mb) are definitely not included into the interval of B1 gene recently cloned (from 681.5 to 706.8 Mb; DeWitt *et al.*, 2020).

Since the remaining regions detected in this study were not reported in previous GWAS works, we hypothesize that most of the MTAs observed in this study, and shown in Table 3, define new and undescribed regions controlling the einkorn traits analyzed. Moreover, several markers, including both mapped and un-mapped, resulted associated to multiple traits (Supplementary Table S4_d), suggesting possible pleiotropic effects of the associated QTL. It would be interesting to investigate deeper these overlapping, especially HDA-SPS and PLH-SPL, trait-association already described in bread wheat (Chai *et al.* 2018; Chen *et al.* 2020) Overall, these observations deserve further investigations and could be exploited both in the breeding of this crop and to transfer valuable alleles into more widespread species such as bread wheat.

## Conclusion

An approach based on co-linearity with bread wheat was adopted to achieve a set of einkorn molecular markers with an inferred physical position; these, together with the phenotypic information from a 4 years field trial experiment, allowed to undertake an association mapping survey on a species poorly characterized from the genetic point of view. Indeed, this is the first report of a GWAS approach on einkorn using a high number of molecular markers and an extensive panel of landraces. The analyses yielded several einkorn genome regions putatively involved in the regulation of phenological, morphological, quality, and production-related traits, which deserve a deeper characterization from the functional point of view. For seven associated regions, we also found confirmation of our results in the literature on bread wheat, despite the remarkable genetic distance between the two species, and this further validates the robustness of the method. Starting from this information, a set of specific markers such as Cleaved Amplified Polymorphic Sequences (CAPSs), or markers amenable for automation and high-throughput approaches (such as Kompetitive Allele Specific PCR: KASPar markers) may be developed and used as valuable tools for the marker-assisted breeding of einkorn wheat.

Unfortunately, no consideration can be done for many other markers, despite significant association *P-*values, because they were not mapped on the bread wheat genome, and only few could be positioned either on *T. urartu* genome (18) or einkorn contigs (48) (Supplementary Table S4_c). Nevertheless, it should be kept in mind that we adopted very stringent mapping criteria, which allowed a very accurate positioning of the markers but, on the other hand, led to define the most part of these as “unmapped”. Despite this, we found several markers influencing multiple traits (*e.g.*, heading date with number of spikelets per spike, and spike length with plant height), and these co-regulation mechanisms found support in literature further validating our analysis. Moreover, when the mapping criteria were loosened, further associations could be mapped and found consistency with literature (data not presented).

The alignments performed to the three species let us to identify 3290 einkorn contigs linked to 3655 markers, out of them 1166 and 1412 were physically mapped on *T. urartu and T. aestivum* genomes, respectively. These results revealed a possible future strategy for ordering and orienting the einkorn contigs currently available but not yet anchored: the results of the BLAST alignments (maybe loosening selection criteria) performed for the whole marker dataset (also including those not passing the callrate and minor allele frequency thresholds adopted for the association mapping) coupled with the available genetic information could be exploited for this purpose.

This would boost the molecular study of still unexplored traits and unlock the efficient use of einkorn as a valuable repertoire of genetic alleles for the breeding of modern crops.

## Authors contributions

A.V. performed the analysis of population structure and GWAS, wrote the first manuscript draft and managed the revision process. D.B. performed the blast of the DArT markers against the wheat genomes (*T. aestivum*, *T. urartu*, and *T. monococcum*) and contributed to manuscript writing. A.V. and D.B. investigated the novelty of the MTAs identified. R.M. contributed to the statistical analyses, to writing and critically revising the manuscript. A.B. conceived and supervised the work, managed in-field trials and phenotyping and revised the manuscript. All the authors read and approved the final manuscript. A.V. and D.B. contributed equally to the paper.

## Code availability (software application or custom code)

The original codes cited in the reference, when specified, were used.

## Data availability

Supplemental files available at figshare: https://doi.org/10.25387/g3.14975118. Table S1 includes the list of the accessions tested, their origin and the cluster assigned by the Structure analysis. Table S2 shows the physical position on bread wheat genome (IWGSC RefSeq V1.0) assigned to DArT markers of *Triticum monococcum.* Table S3 shows the results of the Genome-Wide Association Mapping analysis for markers unmapped on bread wheat genome. Table S4 shows the physical position of DArT markers on *T. urartu* genome (a; GCA_003073225.1) and *T. monococcum* contigs (b; TGAC WGS monococcum v1). In the third sheet (c), the physical position of the peak marker-trait associations (both mapped on bread wheat genome and unmapped) is reported. In the fourth sheet (d), markers associated to multiple traits are listed together with their physical position on the three considered genomes.

File S1 reports the Analysis Of Variance (ANOVA) of the phenotypic data. File S2 contains the genotypic data (DArTseq) of the einkorn panel used for the analysis of population structure, analysis of linkage disequilibrium decay and association mapping. Only the markers passing the callrate (90%) and Minor Allele Frequency (5%) thresholds are shown. File S3 shows the Manhattan- and QQ plot resulting from the association analyses for all the considered traits.
